# ^68^Ga-P15-041, A Novel Bone Imaging Agent for Diagnosis of Bone Metastases

**DOI:** 10.3389/fonc.2021.766851

**Published:** 2021-11-25

**Authors:** Rui Guo, Xiangxi Meng, Fei Wang, Jiangyuan Yu, Qing Xie, Wei Zhao, Lin Zhu, Hank F. Kung, Zhi Yang, Nan Li

**Affiliations:** ^1^ Key Laboratory of Carcinogenesis and Translational Research, (Ministry of Education, Beijing), NMPA Key Laboratory for Research and Evaluation of Radiopharmaceuticals (National Medical Products Administration), Department of Nuclear Medicine, Peking University Cancer Hospital & Institute, Beijing, China; ^2^ Key Laboratory of Radiopharmaceuticals, Ministry of Education, College of Chemistry, Beijing Normal University, Beijing, China; ^3^ Department of Radiology, University of Pennsylvania, Philadelphia, PA, United States

**Keywords:** PET/CT, bisphosphonate, cancer, bone metastases, SUV and Gallium-68

## Abstract

**Objectives:**

^68^Ga-P15-041 (^68^Ga-HBED-CC-BP) is a novel bone-seeking PET radiotracer, which can be readily prepared by using a simple kit formulation and an in-house ^68^Ga/^68^Ge generator. The aim of this study is to assess the potential human application of ^68^Ga-P15-041 for clinical PET/CT imaging and to compare its efficacy to detect bone metastases of different cancers with ^99^mTc-MDP whole-body bone scintigraphy (WBBS).

**Methods:**

Initial kinetic study using Patlak analysis and parametric maps were performed in five histopathologically proven cancer patients (three males, two females) using ^68^Ga-P15-041 PET/CT scan only. Another group of 51 histopathologically proven cancer patients (22 males, 29 females) underwent both ^99^mTc-MDP WBBS and ^68^Ga-P15-041 PET/CT scans within a week, sequentially. Using either pathology examination or follow-up CT or MRI scans as the gold standard, the diagnostic efficacy and receiver operating characteristic curve (ROC) of the two methods in identifying bone metastases were compared (p <0.05, statistically significant).

**Results:**

Fifty-one patients were imaged, and 174 bone metastatic sites were identified. ^68^Ga-P15-041 PET/CT and ^99^mTc-MDP WBBS detected 162 and 81 metastases, respectively. Sensitivity, specificity, positive predictive value, negative predictive value and accuracy of ^68^Ga-P15-041 PET/CT and ^99^mTc-MDP WBBS were 93.1% *vs* 81.8%, 89.8% *vs* 90.7%, 77.5% *vs* 69.2%, 97.2% *vs* 93.4% and 90.7% *vs* 88.4%, respectively. Our results showed that the mean of SUVmax was significantly higher in metastases than that in benign lesions, 15.1 ± 6.9 *vs*. 5.6 ± 1.3 (P <0.001). Using SUVmax = 7.6 as the cut-off value by PET/CT, it was possible to predict the occurrence of metastases (AUC = 0.976; P <0.001; 95% CI: 0.946–0.999). However, it was impossible to distinguish osteoblastic bone metastases from osteolytic bone lesions. Parametric maps based on Patlak analysis provided excellent images and highly valuable quantitative information.

**Conclusions:**

^68^Ga-P15-041 PET/CT, offering a rapid bone scan and high contrast images in minutes, is superior to the current method of choice in detecting bone metastases. It is reasonable to suggest that ^68^Ga-P15-041 PET/CT could become a valuable routine nuclear medicine procedure in providing excellent images for detecting bone metastases in cancer patients. ^68^Ga-P15-041 could become a valuable addition expanding the collection of ^68^Ga-based routine nuclear medicine procedures where ^18^F fluoride is not currently available.

## 1 Introduction

The bone is the third most common site of metastasis for a wide range of solid tumors, and about 70 and 80% of cancer patients will eventually develop bone metastasis (1–3). Bone metastasis often predicts manifestation of cancer, which lead to poor quality of life and shorter life span. Skeletal related events (SRE) such as bone pain, pathological fracture and hypercalcemia are common complications of bone metastasis, which seriously affect the quality of life of patients (4–6). The purpose of bone imaging was to identify bone involvement as early as possible to prevent complications including fractures and spinal cord compression, monitor treatment responses, and guide histological biopsies.

Methylene diphosphonate labeled with technetium-99m (^99m^Tc-MDP) contains the smallest bisphosphonate chelator, and is one of the most commonly used radiopharmaceutical imaging agents over the past forty years. Due to its advantage of overall high sensitivity and easy evaluation of the entire skeleton at a relatively low cost in comparison to conventional radiographs, ^99m^Tc-MDP whole body bone scan (WBBS) has become the most common method for screening bone metastasis. But there are several disadvantages associated with this technique, such as low specificity, hard to distinguish between osteogenic and osteolytic lesions, and not showing the degree of bone destruction (7–9). In addition, there have been few improvements in radiopharmaceuticals for WBBS in the past few decades, and the supply of ^99m^Tc has become less predictable in recent years due to the decline in the number of active nuclear reactors for medical isotope production. However, with the rapid development of positron emission tomography/computed tomography (PET/CT), the role of positron tracer in the detection of bone lesions has attracted more attention as a potential alternative method. When WBBS fails to provide sufficient information for diagnosis, PET/CT may become the game-changing clinical tool to address this problem. Compared with WBBS, PET/CT provides higher resolution bone images, thus it could detect lesions not readily detectable by WBBS. This method might be more suitable for the detection of bone metastases (10–14), and it might have important clinical significance for guiding the selection of tumor treatment and prognosis on patient management. The combined information provided by PET/CT fusion scans not only has advantages in identifying malignant and benign lesions, but also reduces the need for additional imaging procedures, thus avoiding possible diagnostic delays. With the improvement of PET/CT equipment resulting in higher spatial resolution, image quality, multidimensional information and anatomical localization have led to better images for diagnosis and improvement of patient management.

^68^Ga-P15-041 is a novel bone-seeking radiotracer, which provides bone PET/CT imaging agents without the need for a near-by cyclotron (15, 16). The imaging agent is a combination of a gallium-68-chelating bifunctional agent, *N*,*N*’-bis(2-hydroxybenzyl)ethylenediamine-*N*,*N*’-diacetic acid (HBED), and a bone-targeting group—bisphosphonate (BP). Thus, ^68^Ga-P15-041 not only ensures a high specificity through the excellent bisphosphonate binding of active bone surfaces, but also affords a quick and facile radionuclide, gallium-68, complex formation for the diagnosis of bone lesions. Previous report by Zha et al. (15) has demonstrated that biodistribution and microPET imaging studies of ^68^Ga-P15-041 in normal mice and rats showed excellent *in vivo* stability, high bone uptake and retention comparable to that of ^18^F-NaF. Recently, ^68^Ga-P15-041 PET/CT imaging studies in humans was reported by Doot et al. (16). Results showed a higher contrast with more detectable lesions than the planar bone imaging with ^99m^Tc-MDP which demonstrated the potential as a new type of positron tracer for bone imaging. In this study we further evaluate the ability of using maximum standardized uptake value (SUVmax) for measuring the sensitivity, specificity, positive and negative predictive value (PPV and NPV), and accuracy of ^68^Ga-P15-041 PET/CT images to detect bone metastases in cancer patients.

## 2 Materials and Methods

### 2.1 Production of 68Ga-P15-041 (^68^Ga-HBED-CC-BP)

P15-041 was obtained as a lyophilized kit (provided by Professor Lin Zhu from Beijing Normal University). Radiosynthesis, quality control, and final human dose release criteria were performed according to previously reported procedures (15–17).

### 2.2 Subjects

The clinical protocol was reviewed and approved by the Ethics Committee of Peking University Cancer Hospital (ID. 2018KT50) and it was conducted according to the latest guidelines of the Declaration of Helsinki. All patients provided a written informed consent before study participation. The inclusion criteria included the following: older than 18 years; with the ability to provide informed written consent; and with pathological diagnosis of malignant tumor. The exclusion criteria included any of the following: liver and/or renal dysfunction, pregnancy or current lactation, and inability to assume a supine position continuously on the scanner bed. Finally, 56 patients were enrolled, five patients (three males, two females, age 40–67 y, average age 54 y) underwent ^68^Ga-P15-041 dynamic PET/CT imaging, and 51 patients (22 males, 29 females, age 27–84 y, average age 56 y) underwent both ^68^Ga-P15-041 PET/CT imaging and ^99m^Tc-MDP WBBS, sequentially within a week.

### 2.3 Bone Scintigraphy Protocol

No specific preparation was required for patients. The intravenously administered dose of ^99m^Tc-MDP was 740 to 925 MBq. The patients were instructed to empty the bladder 3 h after receiving ^99m^Tc-MDP and planar images were obtained. Planar images were acquired on a dual-head gamma camera (Siemens, Erlangen, Germany) fitted with a low-energy high-resolution parallel hole collimator, at the acquisition speed of 15 cm/min, with a 20% energy window centered at 140 keV. Data acquired were stored in a 256 × 1,024 matrix.

### 2.4 ^68^Ga-P15-041 PET/CT Protocol

Similarly, no specific preparation was required for patients. An intravenously administered dose of ^68^Ga-P15-041 (157–267 MBq) was used. The specific activity of ^68^Ga-P15-041 of each patient was shown in **Supplementary Table 1**. PET/CT scans were conducted by a Siemens Biograph mCT Flow 64 scanner (Siemens, Erlangen, Germany).

Dynamic PET/CT imaging procedures were performed by the following protocol: A low-dose CT scan (120 kV, 35 mA, slice 3 mm) was first performed, and dynamic PET acquisitions started upon the injection of the tracer. An optimized dynamic whole-body acquisition enabled by FlowMotion was conducted over a duration of approximately 60 min (collection speed from fast to slow, 10 passes). Subsequently, a whole-body PET/CT scan was performed in 120 min after the injection, and the acquisition speed was 1.0 mm/s to cover the whole body (slice 3 mm, filter: Gaussian, FWHM: 5 mm), which lasted approximately 15 min. Static PET/CT imaging was performed in 60 min after ^68^Ga-P15-041 injection. The scans covered the length from the top of the skull to the feet, and the acquisition parameters were the same as the 120 min acquisition of dynamic imaging.

Both dynamic and static PET images were reconstructed using a three-dimensional iterative reconstruction with the time-of-flight algorithm, and the low-dose CT scans were acquired in CARE Dose 4D mode (120 kV, 3.0 mm/slice). The dynamic PET scans were segmented and reconstructed into 10 frames.

### 2.5 Parametric Map

According to the time-activity map, it is reasonably assumed that the uptake of the tracer by the lesion is non-reversible. Thus, the dynamic process of tracer uptake could be modeled with the Patlak analysis. In this study, the parametric map was generated for each patient, revealing the Patlak *K_i_* and *V_b_* (3). The input function was retrieved from the images, by manually segmenting the ascending aorta of each patient. A code developed with MATLAB 2020b (Mathworks, MA, USA) was used to generate the images.

### 2.6 Image Analysis

A Siemens workstation (MultiModality Workplace) was used for post processing. Two experienced nuclear medicine/radiologists reviewed and analyzed the rests of ^99m^Tc-MDP WBBS and ^68^Ga-P15-041 PET/CT independently, and any inconsistencies were resolved by consensus.

The standard protocol for bone imaging with ^99m^Tc-MDP WBBS was performed. Abnormal increases in the uptake of ^99m^Tc-MDP in WBBS associated with metastatic bone lesions were identified, if they were not periarticular area involving the posterior vertebral body and pedicle, or rib lesions presented as elongated uptake (18–20).

Differential diagnosis of bone metastasis and benign lesions was based on the uptake intensity and the tomodensitometry characteristics of the lesions observed in the CT component of the PET/CT. Volumes of interest (VOIs) were manually drawn for each lesion and the SUVmax values were automatically calculated. The axial, coronal, and sagittal PET/CT images of ^68^Ga-P15-041 were qualitatively analyzed by nuclear medicine physicians. All of diagnostic criteria have been adapted based on previously published literatures on the diagnosis of bone metastases with ^18^F-NaF PET/CT (18, 21–23). Areas with ^68^Ga-P15-041 uptake higher than normal bone were identified as abnormal, suggesting the presence of bone lesions. Vertebral lesions involving the vertebral body and the posterior pedicle or extensive involvement of the vertebral body are considered malignant. Lesions in the ribs are classified as malignant when they present as strips of high uptake and benign (fractures) when they involve multiple locations of ribs vertically. According to the corresponding morphological characteristics of PET/CT and the anatomical location provided, when degenerative changes, fractures or other benign bone lesions (such as bone cysts) are found at the corresponding location on CT, the lesions are characterized as benign lesions. If lesions associated with osteogenic changes are identified by CT, the lesions are characterized as metastatic. When the local bone was accompanied by abnormal radioactivity concentration without obvious abnormal density changes on CT, it was considered as bone metastasis.

Although histopathological confirmation is considered the golden standard for detecting bone metastases, it was virtually impossible to obtain the information for all lesions in this study. Where feasible, metastatic bone invasion is confirmed by histological examination. Alternatively, clinical follow-up and/or CT/MRI/bone scans were used to confirm the presence of metastasis. For each patient, the presence or absence of bone metastases is determined by combining his/her clinical, radiographic, and biopsy pathological results. Metastatic bone lesions are considered positive, if any of the following criteria are present: positive pathology; other imaging tests (MRI or CT); or progression of skeletal lesions on subsequent imaging or nuclear medicine studies (CT, MRI, BS or ^18^F-FDG-PET) in 6 to 12 months after the initial scan. According to the characteristics of density changes identified by CT, bone metastatic lesions were divided into osteolytic and osteogenic lesions.

In the dynamic imaging study, all metastatic lesions were divided into four regions: spine (including the whole vertebral column), pelvis (including the iliac, ischial and pubic bones), thorax and head (including ribs, scapula and skull bones), and the extremities. To analyze the biodistribution and variability of ^68^Ga-P15-041, VOIs were manually drawn for each metastastic lesion, as well as two benign lesions, fourth lumbar vertebral, liver and right gluteus maximus on every pass, while avoiding major blood vessels. The software automatically calculated SUVmax for the VOIs and the time–activity curves were produced accordingly.

### 2.7 Statistical Analysis

The quantitative data were presented as mean ± standard deviation (SD) and the qualitative data as n (%). Analysis of variance (ANOVA) using repeated measurements was used to compare the variation trend of SUV values in each group of lesions in the dynamic images. Bonferroni method was used to correct P values for multiple comparisons. Sensitivity, specificity, accuracy, PPV and NPV of ^99m^Tc-MDP WBBS and ^68^Ga-P15-041 PET/CT were calculated. The independent Student’s t-test was used for comparison of quantitative variables between two different groups. For paired comparisons of quantitative variables, the paired Student’s t-test was used. A p-value less than 0.05 was considered statistically significant for statistical tests performed. To assess the predictive capacity of the quantitative variables in relation to the occurrence of bone metastasis, the area under the curve (AUC) of the receiver operating characteristic (ROC) was calculated, with a confidence interval (CI) of 95%. SPSS software (version 24, IBM, NY), and Excel software (Microsoft Corporation, Redmond, WA) were used for the statistical analysis of the data.

## 3 Results

### 3.1 ^68^Ga-P15-041 PET/CT Dynamic Imaging

#### 3.1.1 Temporal Characteristic of Tracer Distribution

**Figure 1:**
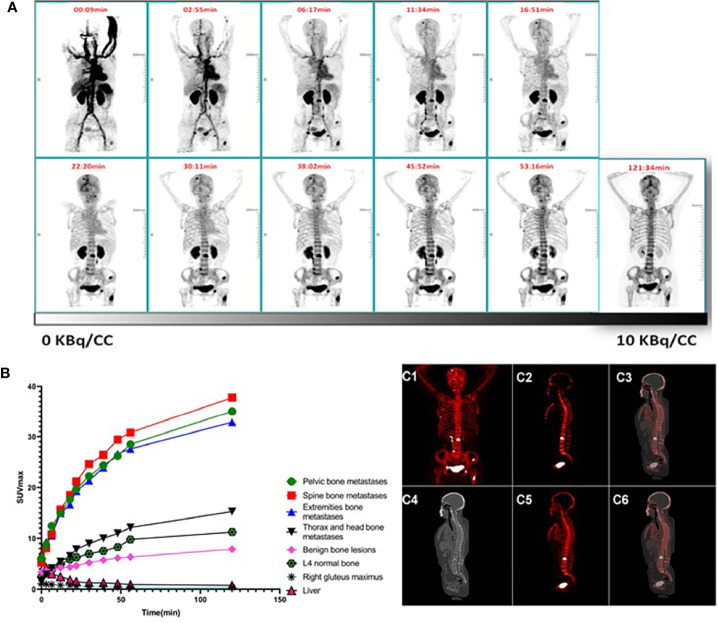
**(A–C)** Pharmacokinetics of ^68^Ga-P15-04.1**(A)** Dynamic Maximum-intensity projections (MIP) of patient #3 who received 220.5 MBq ^68^Ga-P15-041. Top row indicates starting time (min) of whole-body scan. **(B)** Kinetic curves for SUVmax of different sites and lesions with time. Rapid uptake in bone lesions suggest that optimal images may be obtained at early time points. **(C)** Patlak maps and static SUV image of a representative patient (Same patient as in panel **A**). **(C1, C2)** The MIP and a sagittal slice of the Patlak map; **(C3)** the merged image of the Patlak map and CT; **(C4)** the corresponding sagittal CT slice; **(C5, C6)** the corresponding sagittal slice of the routine PET and fused PET/CT.

The clinical information of the five patients receiving the dynamic PET scans is summarized in **Table 1**. A typical case is shown in **Figure 1A**. The time–activity curves of bone metastases, benign bone lesions, liver, L4 vertebral and right gluteus maximus as a function of time after radiotracer injection are shown in **Figure 1B**. Rapid kinetic curves for lesion uptake suggest that bone lesions may be detected within 1 h after in injection. Optimal images may be obtained at early time points. Increased uptakes were observed in the L4 vertebral right gluteus maximus where a relatively lower liver activity was found comparing with bone metastases. Liver and right gluteus maximus activity gradually reduced to a stable level at later time points, while the activity of L4 vertebral with prolonged slow rise to stable level. The activity of bone metastases and bone benign lesions consistently increased over time. In comparison, the SUVmax values of spinal bone, pelvic bone and extremities bone metastases were statistically different from those of benign bone lesions, liver, L4 vertebral and right gluteus maximus (P <0.05). In contrast, the SUVmax values of the thorax and head metastases were not statistically different from those of benign bone lesions (P = 0.893), but they were statistically different from those of liver, muscle and normal bone (P <0.05).

**Table 1:** Information of Patients Enrolled in ^68^Ga-P15-041 PET/CT Dynamic Imaging.

No.	Gender	Age (years)	Diagnosis	Weight (kg)	Dose (MBq)
1	Male	67	Prostate cancer	70	193.1
2	Female	49	Lung cancer	72	208.3
3	Female	55	Lung cancer	74	220.5
4	Male	40	gastric cancer	65	185.0
5	Male	59	colon cancer	68	189.1

#### 3.1.2 The Parametric Map

Patlak maps of the five patients were generated, and the representative K_i_ map is shown in **Figure 1C** as the maximum intensity projection. As demonstrated by the Patlak map, the K_i_ parameter was specifically increased on the lesion sites (0.12 ± 0.013), indicating preferentially and non-reversibly uptake of the tracer. The whole skeleton displayed an elevated, positive K_i_, while the bladder and the ureter showed extremely high parametric values.

### 3.2 Comparison Between ^68^Ga-P15-041 PET/CT and ^99^mTc-MDP WBBS

#### 3.2.1 Patient-Based Analysis

A total of fifty-one tumor patients underwent ^99m^Tc-MDP WBBS and ^68^Ga-P15-041 PET/CT examination within one week, and their clinical information is shown in **Table 2**.

**Table 2:** Clinical Characteristics of Participants for Comparison Study between ^68^Ga-P15-041 PET/CT and ^99m^Tc-MDP WBBS.

Characteristic	Value
No. of patients	51
Male-to-female ratio	1.0:1.32 (22:29)
Mean age ± SD, years (range)	56 ± 10 (27–84)
Primary tumor types No. (%)	
Lung cancer	22 (43.14%)
Breast cancer	14 (27.45%)
Melanoma	4 (7.84%)
Intestinal cancer	4 (7.84%)
Prostate cancer	3 (5.88%)
Renal cancer	2 (3.92%)
Hepatocarcinoma	1 (1.96%)
Nasopharyngeal Cancer	1 (1.96%)

**Figure 2:**
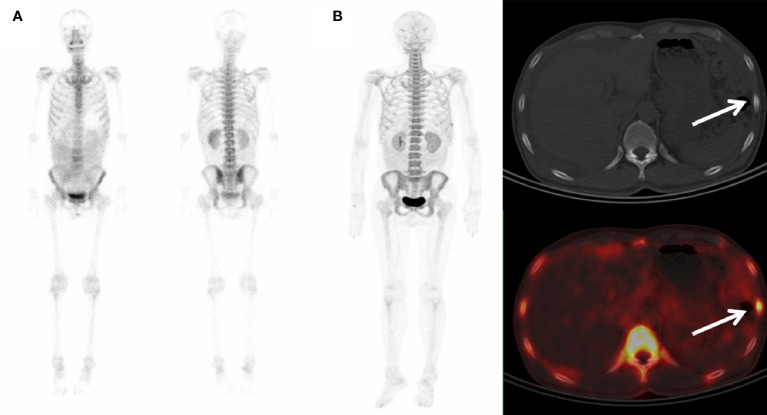
^99m^Tc-MDP WBBS and ^68^Ga-P15-041 PET/CT in a colon cancer patient. **(A)** ^99m^Tc-MDP WBBS appeared to show no abnormal uptake. **(B)** MIP of ^68^Ga-P15-041 and the axial fused PET/CT showed moderate uptake in left 7th rib (arrow) with an osteoblastic change on the axial CT (arrow).

Of the fifty-one patients studied, 47 showed abnormal tracer uptake on ^68^Ga-P15-041 PET/CT, and 45 (45/47, 97.8%) of them were finally diagnosed as bone metastases based on biopsy and imaging follow-up. Among them, 12 patients displayed single bone metastases and 43 patients exhibited multiple bone metastases. In 42 of 45 patients, PET/CT clearly found malignant bone invasion, and bone lesions with increased uptake of ^68^Ga-P15-041 were identified by PET scans with corresponding changes in bone density observed by CT scans. An example is shown in **Figure 2**. Although three patients showed a higher bone uptake without changes in CT morphology on spine and pelvis, these patients were later confirmed by the follow-up CT showing a new high-density lesion colocalized with the high tracer uptake. Two patients, who were suspected of bone metastasis with lower uptake, but showed no changes in the follow-up CT. They were reported as false positives of ^68^Ga-P15-041 PET/CT. Four patients showed no metastases by follow-ups. ^99m^Tc-MDP WBBS correctly detected 39 of 47 patients with metastases and three patients without metastases, and all these patients were correctly diagnosed with ^68^Ga-P15-041 PET/CT. Three patients showed false-positive and six patients showed false-negative. The final diagnosis by PET/CT and WBBS was concordant for 44 (86.3%) patients (39 true positive, three true negative, and two false positive) and discordant for seven (13.7%) patients. The sensitivity, specificity, PPV, NPV and accuracy of all the two imaging techniques for detection of bone metastases of patients are shown in **Tables 3**, **4**.

**Table 3:** Patient-based and Lesion-based Metastases Detection on ^68^Ga-P15-041 PET/CT and ^99m^Tc-MDP WBBS.

By patients					
Group	Patients	[^68^Ga]Ga-P15-041		^99m^Tc-MDP	
		+	−	+	−
metastases	45	45	0	39	6
no metastases	6	2	4	3	3
**By lesions**					
**Group**	**Lesions**	**[^68^Ga]Ga-P15-041**		**^99m^Tc-MDP**	
		**+**	**−**	**+**	**−**
metastases	174	162	12	81	18
no metastases	462	47	415	36	254

**Table 4:** Comparison of Bone Metastases Detection Efficiency Between ^68^Ga-P15-041 PET/CT and ^99m^Tc-MDP WBBS Based by Patients and by Lesions.

Diagnostic efficiency	By patients			By lesions		
	[^68^Ga]Ga-P15-041	^99m^Tc-MDP	P	[^68^Ga]Ga-P15-041	^99m^Tc-MDP	P
Sensitivity, %	100.0%	86.7%	<0.05*	93.1%	81.8%	<0.05*
Specificity, %	66.7%	50.0%	>0.999	89.8%	90.7%	=0.34
PPV, %	95.7%	92.9%	=0.664	77.5%	69.2%	=0.1
NPV, %	100.0%	33.3%	=0.07	97.2%	93.4%	<0.05*
Accuracy, %	96.1%	82.4%	=0.051	90.7%	88.4%	<0.05*

PPV, positive predictive value; NPV, negative predictive value. *Statistically significant.

#### 3.2.2 Lesion-Based Analysis

**Figure 3:**
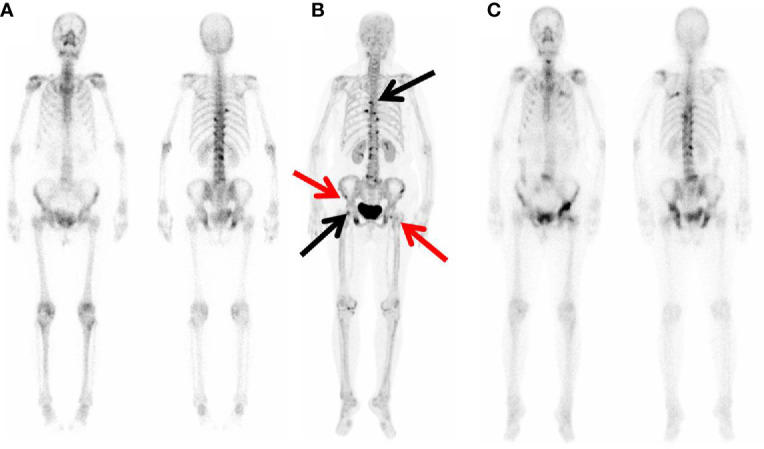
^99m^Tc-MDP WBBS and ^68^Ga-P15-041 MIP in a 72 y patient with Left lung adenocarcinoma. **(A)** ^99m^Tc-MDP WBBS showed multiple high uptake in right 8th rib, left 9th rib, left ilium, bilateral pubic, and thoracolumbar. **(B)** MIP of ^68^Ga-P15-041 showed more high uptake lesions (black and red arrow). **(C)** ^99m^Tc-MDP WBBS after one year later showed disease progression, and right ilium and left femur (black arrow) showed new high uptake.

Finally, 174 bone metastases were confirmed through histopathological and imaging follow-up on 51 patients (**Table 3**). ^68^Ga-P15-041 PET/CT detected 209 (162 true positive; 47 false positive) lesions with bone metastases, and 427 benign lesions (415 true positive; 12 false positive). ^99m^Tc-MDP WBBS alternative was able to detect 117 (81 true positive; 36 false positive) bone metastases and 272 benign lesions (254 true positive; 18 false positive). ^68^Ga-P15-041 PET/CT detected 93.1% (162/174) metastases, corresponding to 3.2 bone metastases (1–24) per patient. However, it appears that ^99m^Tc-MDP WBBS was able only to detect 46.56% (81/174) of the lesions. There was a significant statistical difference between them (*P* < 0.001). An example is shown in **Figure 3**. Sensitivity, specificity, PPV, NPV and accuracy of ^68^Ga-P15-041 PET/CT and ^99m^Tc-MDP WBBS were 93.1% *vs* 81.8%, 89.8% *vs* 90.7%, 77.5% *vs* 69.2%, 97.2% *vs* 93.4% and 90.7% *vs* 88.4%, respectively (**Table 4**). There were 12 false negative lesions mainly small osteolytic metastases, and there were 47 false-positive lesions observed in several high-density foci accompanied by increased tracer uptake.

Locations and final diagnosis of 162 lesions with increased ^68^Ga-P15-041 PET/CT uptake are summarized in **Table 5**. Most bone metastases were detected in the spine skeleton, while the extremities have the least.

**Table 5:** Location and Number Diagnosis of the Skeletal Lesions in ^68^Ga-P15-041 PET/CT and ^99m^Tc-MDP WBBS.

Body Regions	^68^Ga-P15-041 PET/CT			^99m^Tc-MDP WBBS			P
	Putative True P/T (%)			Putative True P/T (%)			
Spine	94	75	79.8%	56	38	67.9%	0.101
Pelvis	56	47	83.9%	27	19	70.4%	0.091
Thorax and head	46	29	63.0%	26	16	61.5%	0.899
Extremities	13	11	84.6%	8	8	100%	0.505
Total	209	162	77.5%	117	81	69.2%	0.1

### 3.3 Analysis of ^68^Ga-P15-041 Uptake of Bone Metastases and Benign Lesions

**Figure 4:**
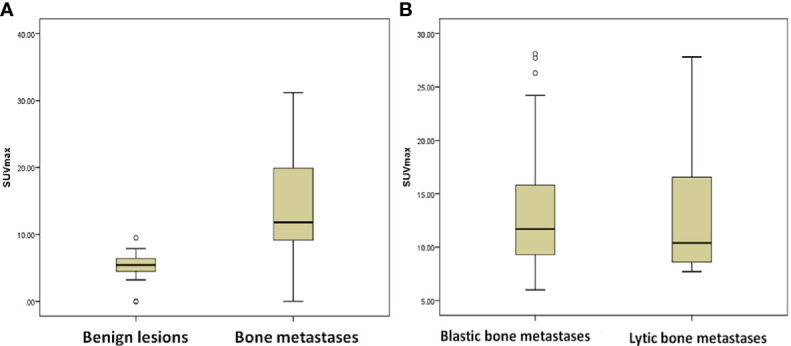
Comparison of SUVmax between different groups in ^68^Ga-P15-041 PET/CT. **(A)** 162 bone metastases and 415 benign lesions. **(B)** Approximately 72 osteoblastic bone metastases and 82 osteolytic bone metastases.

**Figure 5:**
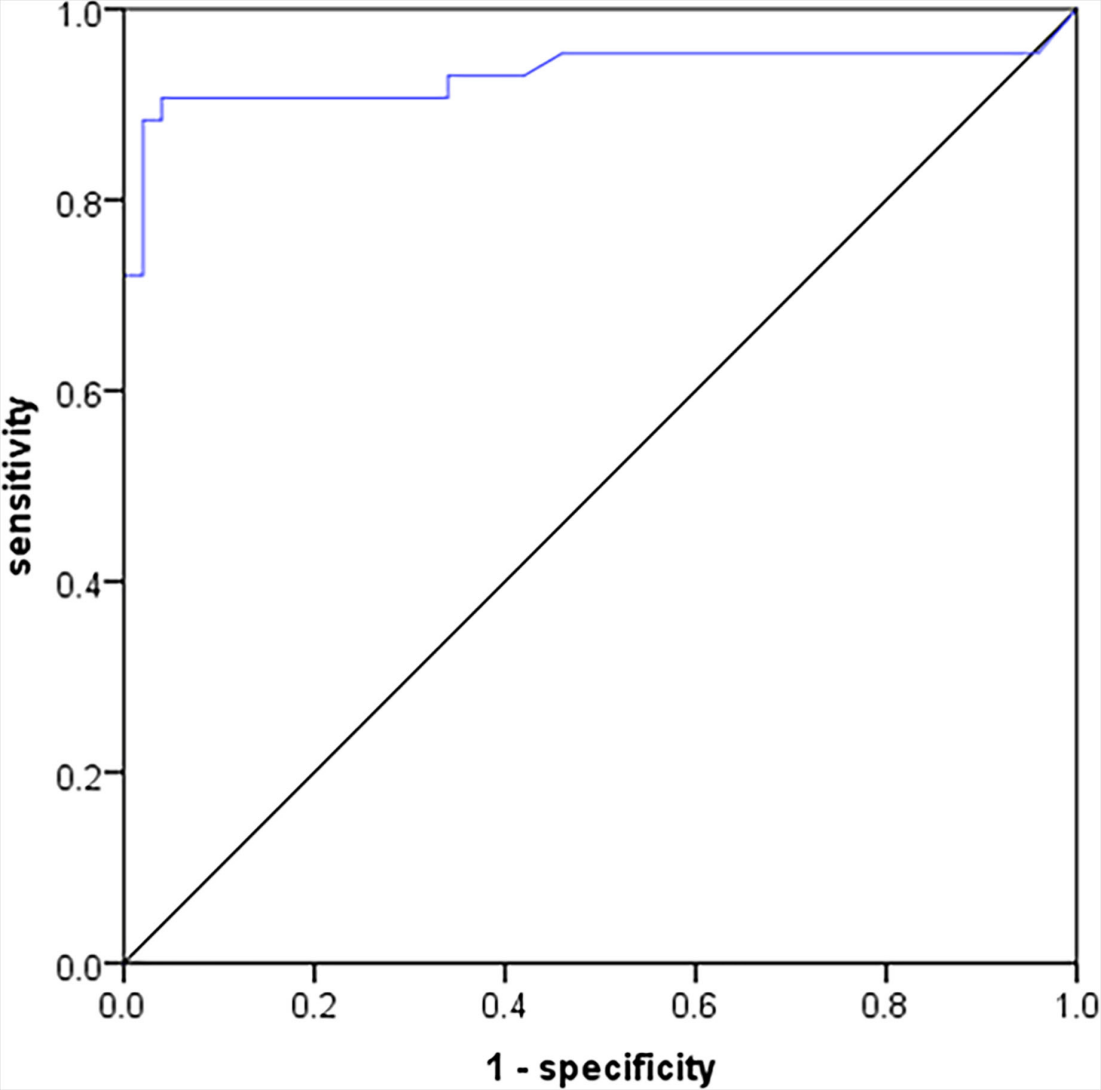
ROC of SUVmax in ^68^Ga-P15-041 PET/CT to discriminate bone metastases from benign lesions.

The SUVmax of the lesions were recorded and compared between 162 bone metastases and 415 benign bone lesions in the ^68^Ga-P15-041 PET/CT. The mean of the SUVmax values was significantly higher in bone metastases than in benign lesions (15.1 ± 6.9 *vs*. 5.6 ± 1.3, *P* < 0.001) (**Figure 4A**). Using the SUVmax, it was possible to predict the occurrence of bone metastases, with the AUC of 0.976 (95%CI: 0.946–0.999), and the sensitivity, specificity, PPV, NPV and accuracy were 95.1, 95.8, 95.1, 95.8, and 95.5%, respectively. An SUVmax above 7.6 always represented bone metastases (**Figure 5**).

Among 162 true positive lesions detected by ^68^Ga-P15-041 PET/CT, 72 lesions (44.4%) showed characteristic osteoblastic metastases with SUVmax of 14.2 ± 6.6; whereas 82 lesions (50.6%) showed osteolytic with SUVmax of 13.7 ± 7.7, and eight lesions (5%) showed no morphology changes on PET/CT. The mean of the SUVmax values was not significantly difference between osteoblastic bone metastases and osteolytic bone metastases (P = 0.887), which was presented in **Figure 4B**. The performances including sensitivity, specificity, PPV, NPV of ^68^Ga-P15-041 PET/CT in different types of bone metastases were listed in **Table 6**.

**Table 6:** Diagnostic efficiency of ^68^Ga-P15-041 in different types of bone metastases.

Diagnosticefficiency	osteoblastic metastases	Osteolytic metastases	Metastases with no morphology changes	P
Sensitivity, %	100.0%	90.1%	72.7%	<0.001*
Specificity, %	90.3%	87.5%	71.4%	=0.357
PPV, %	63.16%	96.5%	80%	<0.001*
NPV, %	100.0%	70%	62.5%	<0.001*
Accuracy, %	91.65%	89.6%	72.2%	=0.057

*Statistically significant.

**Figure 6:**
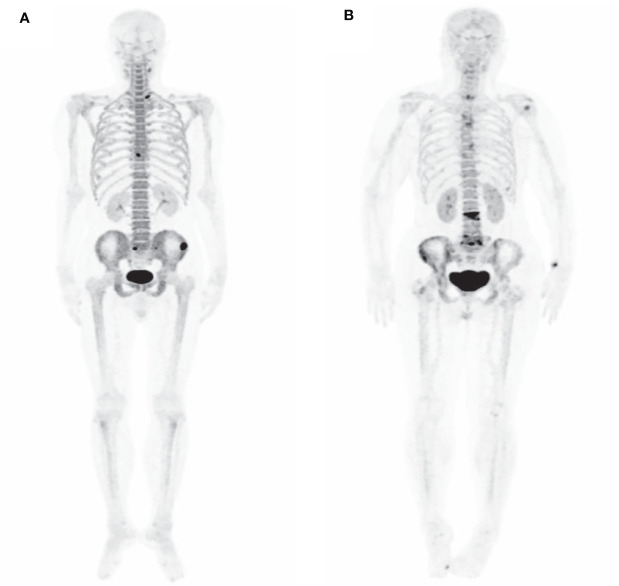
MIP PET images of two patients with multiple bone metastases. **(A)** A 53 y male patient with Left lung adenocarcinoma. **(B)** A 50 y female patient with invasive ductal carcinoma of the left breast.

Among the 51 patients, there were 22 lung cancers, 14 breast cancers and 15 other cancers. The SUVmax of 22 patients with lung cancer and 14 patients with breast cancer were 15.3 ± 7.0 and 14.5 ± 7.4, respectively, showing no statistical difference (p = 0.753). A typical case is shown in **Figure 6**.

## 4 Discussion

Bone metastasis is a major cause of pain and it increases the risk of SRE in cancer patients (4–6). Cancer patients with bone metastases are rarely cured; therefore, accurate diagnosis of bone metastasis is essential for patient management providing information on initial staging, treatment planning, restaging, monitoring, and survival prediction (24–26). At present, ^99m^Tc-MDP WBBS is the most commonly screening method for bone metastasis, but it offers a detecting method with high sensitivity and low specificity. Nevertheless, the limited specificity of WBBS often requires additional examinations to confirm its accuracy, especially in patients with only one single bone metastatic lesion. As an alternative, ^18^F-NaF PET/CT has been shown to be superior to planar bone imaging in the diagnosis of bone metastases. However, ^18^F-NaF PET/CT is not widely available and more expensive, especially in developing countries, and the procedure requires a cyclotron and a team of skilled staff (16, 27). Decades after the introduction of ^99m^Tc-MDP bone scan, it has been evolved into a basic core business for nuclear medicine department. Recent success in using ^68^Ga/^68^Ge generator generator-based ^68^Ga-DOTATATE and ^68^Ga-PSMA-11 for routine diagnosis of neuroendocrine and prostate cancer, respectively, would likely to increase the acceptance of ^68^Ga tracers for routine clinical application. ^68^Ga-P15-041 PET/CT, reported in this paper could become a valuable addition expanding the collection of ^68^Ga-based routine nuclear medicine procedures.

It is known that ^68^Ga based bone targeting tracers can be useful alternative choices because ^68^Ge/^68^Ga generators are relatively inexpensive, readily accessible and easy to operate (28, 29). Noticeably, ^68^Ga-DOTA-ZOL was also reported as a useful for evaluation of bone metastasis (30–32). Synthetic peptides containing the arginine-glycine-aspartate (RGD) sequence motif are active modulators of cell adhesion and can bind specifically to integrin αvβ3. Mi et al. (33) reported ^18^F-Alfatide II PET/CT can be used to detect skeletal and bone marrow metastases, with nearly 100% sensitivity in osteolytic, mixed and bone marrow lesions. The sensitivity of ^18^F-Alfatide II PET/CT in osteoblastic metastases is relatively low but still significantly higher than that of ^18^F-FDG PET/CT. Moreover, the mechanisms of bisphosphonate uptake to active bone surfaces for ^68^Ga-P15-041 are analogous to those of MDP, providing a rationalized comparability of bone metastasis. Therefore, ^68^Ga-P15-041 is a novel bisphosphonate bone-seeking PET imaging agent with relatively low effective dose when compared with ^99m^Tc-MDP and ^18^F-NaF (16).

Results reported for ^68^Ga-P15-041 in a large patient population consisting of both osteoblastic and osteolytic lesions displayed consistent higher uptakes. Adding dynamic imaging with Patlak map was found to be highly effective in assisting the diagnosis. Apart from static acquisition, whole-body dynamic PET imaging better reflects the kinetic aspect of tracer uptake, through continuous acquisition over an extended period of time (34). Different types of parametric maps have been developed to visualize the kinetic parameters based on the dynamic imaging (35). Patlak plot is an important parametric map which was found useful in ^99m^Tc-MDP (36). Compared to the static PET images from which the parametric map was calculated, the Patlak map visualized the metastatic lesions clearly. Due to this enhanced specificity, the Patlak map may enhance the accuracy of diagnosis of bone diseases.

Compared to ^99m^Tc-MDP, patients undergoing ^68^Ga-P15-041 PET/CT required a shorter scanning time (1 h *vs*. 3 h), which may lead to better tolerability and improved patient compliance. Due to the physics of positron emission and *in vivo* kinetics of the tracer, the image quality of ^68^Ga-P15-041 PET/CT is superior to planar bone scintigraphy. Many lesions missed by the ^99m^Tc-MDP WBBS, including bone metastases and benign bone lesions were detected by PET/CT scan. The CT component of ^68^Ga-P15-041 PET/CT can have added advantage of revealing the anatomical morphology and density change of lesions, which is helpful in improving the accuracy of the diagnosis of bone metastases.

Results of ^68^Ga-P15-041 PET/CT scans demonstrated a higher sensitivity, NPV and accuracy for diagnosis of bone metastases than WBBS (sensitivity 93.1% *vs*. 81.8%, NPV 97.2% *vs*. 93.4%, accuracy 90.7% *vs*. 88.4%). There was observable difference in sensitivity and no difference in specificity and accuracy in the patient-based analysis, probably because the diagnosis of bone metastases in patients required only one lesion to be identified. However, the specificity for both tracers showed no statistical difference, and it may be due to the fact that the density and sizes of some osteoblastic lesions did not change in the follow-up CT or MRI scans. Based on our research criteria for the diagnosis, these lesions eventually were diagnosis as false positives. These types of osteoblastic lesions were difficult, if not impossible, to identify by CT or MRI and they usually took longer to follow-up.

Analysis of images by SUVmax was employed as an effective tool in the estimation of the intensity of radiopharmaceutical uptake in the lesion, the evaluation of the disease, and interpatient comparison (14, 37). Published studies have suggested that SUVmax can effectively differentiate the metabolic changes of ^18^F-NaF PET/CT bone lesions, which supplemented by visual qualitative evaluation, can distinguish benign and metastasis lesions (38, 39). Our study also shows that SUVmax was significantly higher in bone metastases than in benign lesions (15.1 ± 6.9 *vs*. 5.6 ± 1.3, *P* <0.001). When the SUVmax is greater than 7.6, it is likely to be recognized as bone metastases, with the sensitivity, specificity and accuracy as high as 95.1, 95.8, and 95.5%, respectively. There were also overlapping SUVmax values between benign lesions and bone metastases. Degenerative bone lesions accompanied by inflammation and certain bone metastases with relatively low metabolism are still difficult to diagnose, it may be rely on MRI to improve the detection. In the future, further prospective studies are needed to establish the role of SUVmax as a semi-quantitative parameter in ^68^Ga-P15-041 PET/CT in accurately identifying benign lesions to skeletal metastatic diseases.

The kinetic data and SUVmax values observed in these patients were very consistent with those previously reported by Doot et al. (16), which confirming the reproducibility of this tracer on detecting metastasis in cancer patients at different nuclear medicine departments. There are several other ^68^Ga labeled bisphosphonates which have appeared in the literature (40–45). No direct comparison of these tracers in cancer patients has been reported. However, based on results included in this paper and other encouraging data presented previously, it is reasonable to suggest that ^68^Ga-P15-041 PET/CT is a candidate worthy of further clinical evaluation.

It is well known that bone metastases were classified into osteoblastic and osteolytic lesions (46, 47). Based on the difference of CT density they may be identified; it was found that osteolytic lesion may have a poorer prognosis and may require different treatments. Calculated SUVmax values based on PET/CT may assist in detecting bone metastases without abnormal density change on CT, which is a useful supplement to CT qualitative assessment. However, there is no significant difference in SUVmax between bone metastases from different primary tumors (lung cancer *vs*. breast cancer) and between osteoblastic and osteolytic lesions. It is likely that partial osteolytic lesions may be accompanied by osteogenic changes, which might affect the SUVmax values observed by PET/CT scans.

Several limitations must be considered in this study. First of all, a great majority of the patients lacked histopathological verification of the bone metastases, resulting to the reliance of imaging follow-up as the standard of reference. This may increase the clinical heterogeneity of patient samples, but further stratification by obtaining histological proof of all skeletal lesions is either impractical or clinically unethical. Therefore, non-invasive imaging examination results that have not been strictly verified by histological examination are acceptable under the current situation. Secondly, despite the statistically significant differences there is an overlap of SUVmax values between bone metastases and benign lesions. Although a large number of patients were analyzed, various cancer categories may lead to increased clinical heterogeneity, and due to limited patient data, we were not able to perform subgroup analysis for each cancer category.

## 5 Conclusion

Results presented in this paper suggest that ^68^Ga-P15-041 in conjunction with PET/CT imaging is suitable for noninvasive detection of bone metastases in cancer patients. Whole body ^68^Ga-P15-041 PET/CT scans are more sensitive and accurate than those of conventional ^99m^Tc-MDP WBBS in detecting bone metastases. Compared to ^99m^Tc-MDP, this imaging method has advantages of being able to perform earlier imaging and providing images with better contrast. Using SUVmax as the key parameter, it can serve as a useful means for the quantification of PET/CT, and it may also improve the differentiation between bone metastases and benign lesions. ^68^Ga-P15-041 in conjunction with PET/CT may serve as a method of choice for diagnosis of bone metastases in cancer patients in the future.

## Data Availability Statement

The raw data supporting the conclusions of this article will be made available by the authors, without undue reservation.

## Ethics Statement

The studies involving human participants were reviewed and approved by the Medical Ethics Committee of Peking University Cancer Hospital. The patients/participants provided their written informed consent to participate in this study.

## Author Contributions

RG, ZY, and NL jointly designed the study and executed the protocols. RG and JY took the major responsibility in the study cohort, and XM in data analysis. WZ assisted the data acquisition. JY and FW were involved in the image evaluation. NL and ZY jointly supervised the whole research process, conceptually designed the research ideas and provided resources. LZ provided the lyophilized kit. QX was in charge of drug synthesis. HK and NL helped draft the manuscript. All authors provided critical feedback and helped shape the research, analysis, and manuscript, and discussed the results. All authors contributed to the article and approved the submitted version.

## Funding

This work was financially supported by the National Natural Science Foundation (No. 81871387; No. 81871386), the Beijing Natural Science Foundation (No. 7202027), and the Beijing Municipal Administration of Hospitals—Yangfan Project (ZYLX201816).

## Conflict of Interest

The authors declare that the research was conducted in the absence of any commercial or financial relationships that could be construed as a potential conflict of interest.

## Publisher’s Note

All claims expressed in this article are solely those of the authors and do not necessarily represent those of their affiliated organizations, or those of the publisher, the editors and the reviewers. Any product that may be evaluated in this article, or claim that may be made by its manufacturer, is not guaranteed or endorsed by the publisher.
