# Supplementary material for: 68Ga-P15-041, A Novel Bone Imaging Agent for Diagnosis of Bone Metastases
Source: Front Oncol. 2021 Nov 25;11:766851. doi: 10.3389/fonc.2021.766851 (PMC8654731; doi:10.3389/fonc.2021.766851)
Supplement: Supplementary file 1 [file Table_1.pdf]

# $^{68}$Ga-P15-041, a novel bone imaging agent for diagnosis of bone metastases

**Supplementary Table 1.** The specific activity of $^{68}$Ga-P15-041 used in each patient

| | Gender | Age(y) | The specific activity of $^{68}$Ga-P15-041(MBq/umol) |
|---|---|---|---|
| Patient1 | Male | 61 | 0.080 |
| Patient2 | Female | 57 | 0.070 |
| Patient3 | Male | 38 | 0.080 |
| Patient4 | Female | 60 | 0.080 |
| Patient5 | Female | 54 | 0.091 |
| Patient6 | Female | 53 | 0.094 |
| Patient7 | Female | 47 | 0.084 |
| Patient8 | Male | 27 | 0.084 |
| Patient9 | Female | 61 | 0.073 |
| Patient10 | Female | 56 | 0.080 |
| Patient11 | Female | 48 | 0.084 |
| Patient12 | Female | 50 | 0.084 |
| Patient13 | Female | 62 | 0.073 |
| Patient14 | Male | 55 | 0.091 |
| Patient15 | Female | 49 | 0.077 |
| Patient16 | Male | 67 | 0.066 |
| Patient17 | Male | 56 | 0.080 |
| Patient18 | Male | 53 | 0.080 |
| Patient19 | Female | 64 | 0.080 |
| Patient20 | Female | 34 | 0.087 |
| Patient21 | Male | 65 | 0.087 |
| Patient22 | Male | 57 | 0.070 |
| Patient23 | Female | 69 | 0.063 |
| Patient24 | Male | 71 | 0.063 |
| Patient25 | Male | 50 | 0.084 |
| Patient26 | Female | 56 | 0.084 |
| Patient27 | Female | 65 | 0.073 |
| Patient28 | Female | 67 | 0.091 |
| Patient29 | Male | 84 | 0.087 |
| Patient30 | Male | 73 | 0.087 |
| Patient31 | Male | 32 | 0.084 |
| Patient32 | Male | 68 | 0.080 |

| | | | |
|---|---|---|---|
| Patient33 | Male | 50 | 0.080 |
| Patient34 | Male | 62 | 0.091 |
| Patient35 | Female | 50 | 0.084 |
| Patient36 | Female | 54 | 0.087 |
| Patient37 | Female | 63 | 0.080 |
| Patient38 | Female | 40 | 0.066 |
| Patient39 | Male | 56 | 0.077 |
| Patient40 | Female | 50 | 0.063 |
| Patient41 | Male | 54 | 0.063 |
| Patient42 | Female | 66 | 0.066 |
| Patient43 | Female | 71 | 0.066 |
| Patient44 | Female | 55 | 0.056 |
| Patient45 | Female | 55 | 0.063 |
| Patient46 | Male | 64 | 0.066 |
| Patient47 | Male | 44 | 0.052 |
| Patient48 | Male | 64 | 0.049 |
| Patient49 | Female | 50 | 0.063 |
| Patient50 | Female | 50 | 0.063 |
| Patient51 | Female | 48 | 0.059 |
